# Fasudil Ameliorates Methotrexate-Induced Hepatotoxicity by Modulation of Redox-Sensitive Signals

**DOI:** 10.3390/ph15111436

**Published:** 2022-11-19

**Authors:** Esam M. Aboubakr, Ahmed R. N. Ibrahim, Fares E. M. Ali, Ahmed A. E. Mourad, Adel M. Ahmad, Amal Hofni

**Affiliations:** 1 Department of Pharmacology and Toxicology, Faculty of Pharmacy, South Valley University, Qena 83523, Egypt; 2 Department of Clinical Pharmacy, College of Pharmacy, King Khalid University, Abha 62529, Saudi Arabia; 3 Department of Biochemistry, Faculty of Pharmacy, Minia University, Minia 61511, Egypt; 4 Department of Pharmacology and Toxicology, Faculty of Pharmacy, Al-Azhar University, Assiut Branch, Assiut 71524, Egypt; 5 Pharmacology and Toxicology Department, Faculty of Pharmacy, Port-Said University, Port-Said 42511, Egypt; 6 Department of Pharmaceutical Analytical Chemistry, Faculty of Pharmacy, South Valley University, Qena 83523, Egypt

**Keywords:** MTX hepatotoxicity, rho-kinase inhibitor, hepatic concentration, RP-HPLC technique

## Abstract

Methotrexate (MTX) is one of the most widely used cytotoxic chemotherapeutic agents, and it is used in the treatment of different autoimmune disorders. However, the clinical applications of MTX are limited by its hepatic toxicity. Hence, the present study was conducted to evaluate the efficacy of fasudil (Rho-Kinase inhibitor) in the amelioration of MTX hepatotoxicity and the possible underlying mechanisms. Experimentally, 32 male Sprague Dawley rats were used and divided into four groups: control, MTX (20 mg/kg, i.p., single dose), fasudil (10 mg/kg/day i.p.) for one week, and fasudil plus MTX. It was found that MTX significantly induced hepatitis and hepatocellular damage, as shown by abnormal histological findings and liver dysfunction (ALT and AST), with up-regulation of the inflammatory mediators NF-κB-p65 and IL-1β. Moreover, MTX remarkably disrupted oxidant/antioxidant status, as evidenced by malondialdehyde (MDA) up-regulation associated with the depletion of superoxide dismutase (SOD), catalase, and reduced glutathione (GSH) levels. Moreover, MTX reduced the hepatic expression of B-cell lymphoma 2 (Bcl-2). On the contrary, the i.p. administration of fasudil significantly ameliorated MTX hepatotoxicity by histopathological improvement, restoring oxidant/antioxidant balance, preventing hepatic inflammation, and improving the hepatic anti-apoptotic capability. Furthermore, fasudil hepatic concentration was determined for the first time using the validated RP-HPLC method. In conclusion, the present study revealed that fasudil has a reliable hepatoprotective effect against MTX hepatotoxicity with underlying antioxidant, anti-inflammatory, and anti-apoptotic mechanisms. It also introduced a new method for the determination of fasudil hepatic tissue concentration using the RP-HPLC technique.

## 1. Introduction

Methotrexate (MTX) acts as an antifolate and antimetabolite, which is used extensively in the treatment of different cancerous diseases [1]. MTX, used in different autoimmune and inflammatory diseases, has shown a potent immunomodulatory effect [2]. MTX acts by blocking dihydrofolate reductase, which inhibits folate metabolism and the synthesis of purines and pyrimidines, resulting in decreased RNA and DNA synthesis. It has been reported that MTX administration leads to serum elevation of aminotransferase and has been linked to the induction of liver diseases such as liver fibrosis and cirrhosis [3].

Different regimens using MTX in humans are classified as high-dose, intermediate, or low-dose according to the type of cancer and route of drug administration [4]. MTX administration in high doses (20 mg/kg) can result in a significant elevation of alanine transaminase (ALT) and aspartate transaminase (AST) serum levels up to 10 to 20 times the upper limits [5]. On the other hand, with long-term administration (1–12 months) of MTX, the development of hepatitis, fatty liver, hepatic fibrosis, portal hypertension, and symptomatic cirrhosis has been demonstrated [6,7,8]. The mechanism of hepatic injury may be related to the accumulation of MTX in tissue and the inhibition of RNA and DNA synthesis in hepatic cells, producing cellular arrest [9]. MTX treatment increases hepatic stellate cells, but the mechanism underlying its fibrotic effect has not been elucidated [10,11].

Various studies have demonstrated the role of reactive oxygen species (ROS) generation in the initiation of MTX hepatotoxicity. Thus, it was found that the excessive generation of reactive oxygen and nitrogen species (ROS/RNS) combined with the reduction in antioxidant defense enzymes promotes the development of hepatocellular damage [12]. Moreover, recent studies have clarified the role of inflammatory mediators such as tumor necrosis factor-alpha (TNF-α) and inducible nitric oxide synthase (iNOS) in mediating MTX hepatocellular damage [13,14,15].

On the other hand, Rho-kinase acts as a molecular control of several important cellular functions, such as actin cytoskeleton organization, ROS formation, cellular adhesion and apoptosis [16,17], cytokinesis and oncogenic transformation, and other factors that may be involved in the pathogenesis of hepatic injury. Interestingly, Rho-kinase inhibitors were effective in the amelioration of reperfusion injury in the liver and heart [18,19]. In addition, accumulated evidence demonstrated that ROS activates the Rho/ROCK pathway, and the inhibition of that pathway produces anti-inflammatory effects [20,21]. Additionally, pleiotropic properties [22,23] such as the anti-oxidant and anti-inflammatory functions of statins are thought to be conducted through the inhibition of the ROCK pathway [19,24]. It has been reported that fasudil exhibited therapeutic potential against liver fibrosis [25,26], cardiac toxicity [27], nephrotoxicity [28], and brain injury [29].

Herein, the present study was conducted to investigate Rho-kinase pathway inhibition using fasudil as a target in the development of a new approach to ameliorate MTX-induced hepatotoxicity. Moreover, a novel method used in the determination of hepatic tissue concentration of fasudil was elucidated.

## 2. Results

### 2.1. Chromatographic Method Validation for Fasudil Tissue Concentration

**Figure 1:**
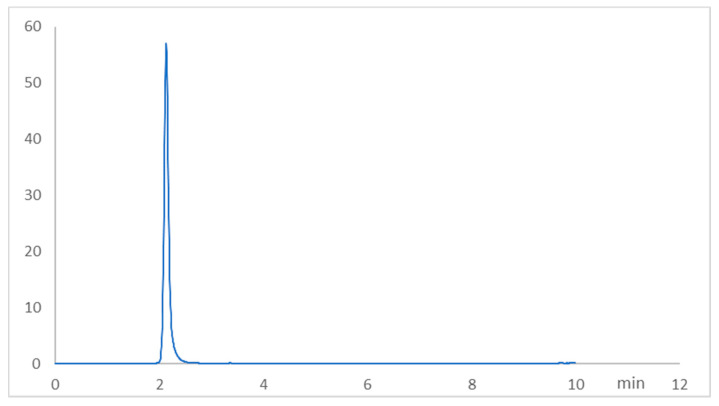
Representative RP-HPLC chromatogram of fasudil (2.12 min, 3.0 μg/mL) under optimized conditions.

To create a sensitive and accurate HPLC method for the quantitation of fasudil, the mobile phase parameters were enhanced to produce symmetric and sharp peaks. After several attempts, an optimum mobile phase was established, which was composed of 15% methanol/85% water containing 10 mM SDS and 20 mM ammonium acetate, and the column temperature was set at 35 °C. The chromatogram is shown in Figure 1, where the retention time of fasudil was obtained at 2.12 min. The developed method was validated concerning ICH guidelines regarding linearity, accuracy, precision, the limit of detection (LOD), and the limit of quantitation (LOQ) (Table 1, Table 2 and Table 3). The system suitability criteria for the developed method such as the tailing factor, asymmetry factor, number of theoretical plates, and the height equivalent to a theoretical plate (HETP) were evaluated (Table 4).

In the present study, the fasudil concentration in the hepatic tissue homogenate was found at 5.32 ± 1.5 µg/g proteins in the fasudil group, while the MTX + fasudil group showed a higher concentration of fasudil as it was found at 6.91 ± 1.33 µg/g proteins. These results suggested that the co-administration of MTX with fasudil significantly (*p* > 0.05) increased the concentration of fasudil in hepatocytes against the administration of fasudil alone.

### 2.2. Effect of Fasudil on Hepatic Enzymes and Lipid Peroxidation after MTX Challenge

**Figure 2:**
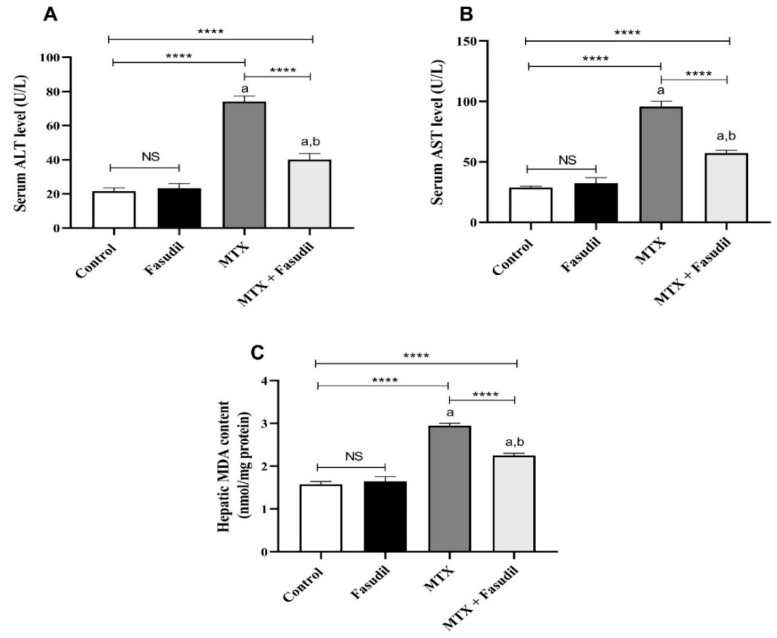
Effect of fasudil on hepatic enzymes and lipid peroxidation after MTX challenge: (**A**) serum ALT; (**B**) serum AST; (**C**) hepatic content of MDA. Results are presented as mean ± SEM (*n* = 8); a: significant difference from the control group (*p* < 0.05); b: significant difference from the MTX control group (*p* < 0.05). **** *p* < 0.0001. NS, non-significant.

The i.p. administration of MTX significantly (*p* < 0.05) induced hepatic injury characterized by marked elevation of the hepatic MDA concentrations (2.95 nmol/mg protein) accompanied by an elevation of the hepatic enzymes ALT (74 U/L) and AST (95.6 U/L) serum concentrations compared to the control group (1.57 nmol/mg protein) for MDA, (21.5 U/L) for ALT, and (28.75 U/L) for AST (Figure 2). However, the concomitant administration of fasudil with MTX significantly (*p* < 0.05) attenuated the MTX hepatic tissue-damaging effect with 2.25 nmol/mg protein for MDA, 40 U/L for ALT, and 57.25 U/L for AST, though the i.p. administration of fasudil for seven consecutive days did not produce significant changes in the formerly mentioned parameters compared to the control group (Figure 2).

### 2.3. Effect of Fasudil on Hepatic Aberrations Induced by MTX Injection

**Figure 3:**
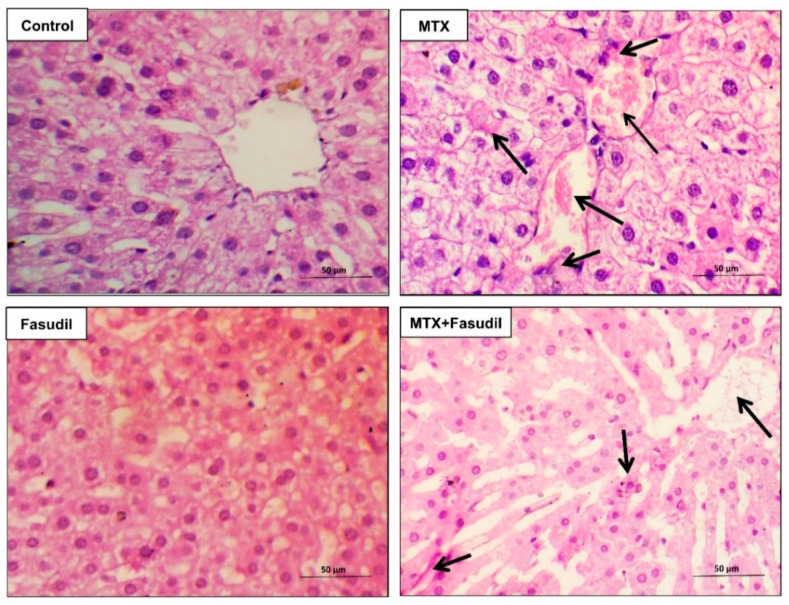
Fasudil ameliorated MTX-induced hepatic histopathological aberrations. Routine staining (hematoxylin and eosin (H&E) stain, ×400) was conducted to examine control, MTX, fasudil, and MTX + fasudil groups. Arrows mark hepatic changes, including hepatocyte degeneration and congestion with inflammatory cell infiltration. Scale bar = 50 μm.

The hepatic tissue of the control and fasudil groups showed normal cellular architecture with hepatocytes in normal arrangement. On the other hand, the hepatic tissue of the MTX group showed different histopathological changes, including hepatic lobular changes with hepatocyte degeneration and congestion with inflammatory cell infiltration. However, the histopathological lesions in MTX + fasudil groups were notably ameliorated compared to the MTX group with mild inflammatory cell infiltration and congestion (Figure 3).

### 2.4. Effect of Fasudil on the Hepatic GSH Content as well as Antioxidant Enzyme Activity

**Figure 4:**
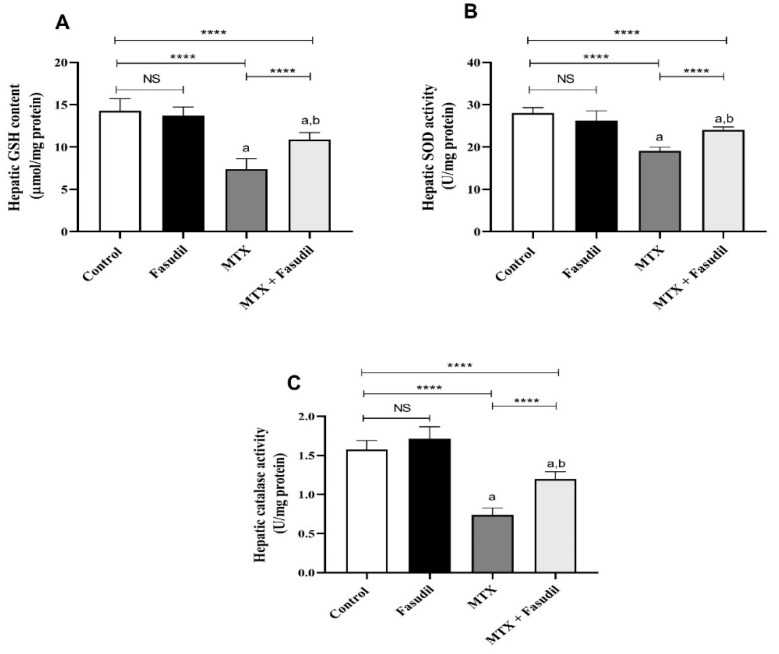
Effect of fasudil on the hepatic GSH content as well as antioxidant enzyme activity: (**A**) hepatic GSH content; (**B**) hepatic SOD activity; (**C**) hepatic catalase activity. Results are presented as mean ± SEM (*n* = 8); a: significant difference from the control group (*p* < 0.05); b: significant difference from the MTX group (*p* < 0.05). **** *p* < 0.0001. NS, non-significant.

The i.p. administration of MTX by a 20 mg/kg dose significantly (*p* < 0.05) decreased the GSH content of the hepatic tissue (7.3 ± 0.45 μmol/mg protein) compared to the control group (14.23 ± 1.33 μmol/mg protein). On the other hand, the i.p. administration of fasudil could restore GSH hepatic contents (10.87 ± 0.98 μmol/mg protein) and ameliorate MTX depleting effects, while the i.p. administration of fasudil to control animals did not produce any significant changes in the hepatic GSH concentrations (Figure 4).

In the present study, the hepatic tissue concentrations of SOD and catalase in the control group were found (28 U/mg protein and 1.57 U/mg protein, respectively), whereas the i.p. administration of MTX produced a significant (*p* < 0.05) reduction of both enzymes to 19.12 U/mg protein and 0.73 U/mg protein, respectively. However, the i.p. administration of fasudil in combination with MTX could significantly attenuate the MTX depleting effect on these antioxidant enzymes concentrations, namely, 19.12 U/mg protein for SOD and 0.73 U/mg protein for catalase (Figure 4).

### 2.5. Effect of Fasudil on IL-1β Expression

**Figure 5:**
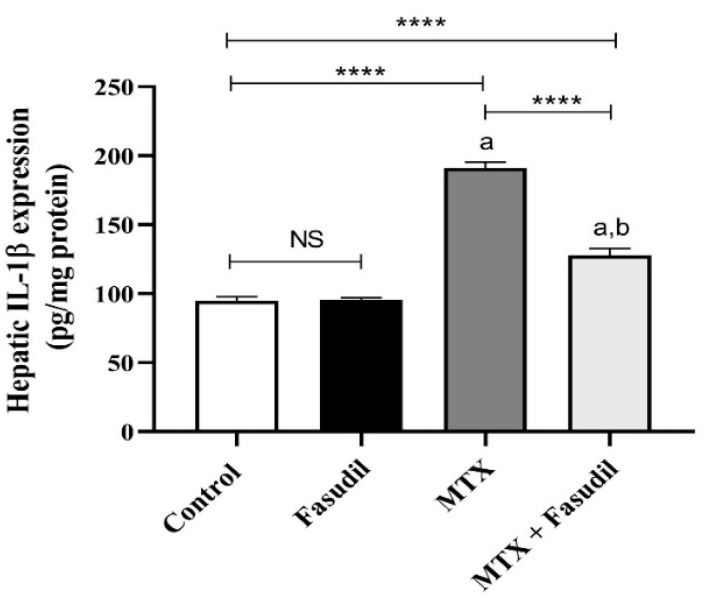
Effect of fasudil on IL-1β expression. Results are presented as mean ± SEM (*n* = 8); a: significant difference from the control group (*p* < 0.05); b: significant difference from the MTX group (*p* < 0.05). **** *p* < 0.0001. NS, non-significant.

The i.p. administration of MTX produced a significant elevation of the inflammatory mediator IL-1β up to 191.12 pg/mg protein compared to 94.87 pg/mg protein for the control group, which was significantly attenuated by i.p. administration of fasudil when combined with MTX (127.87 pg/mg protein), while the i.p. administration of fasudil did not produce any significant change in the IL-1β compared to the control group (Figure 5).

### 2.6. Effect of Fasudil on NF-κB-p65 and Bcl-2 Expressions

**Figure 6:**
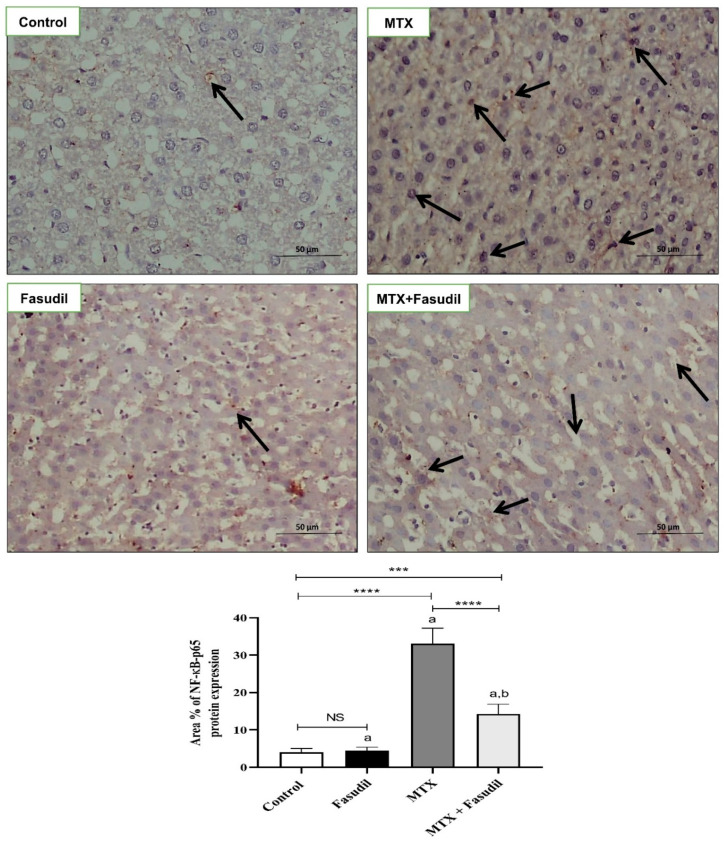
Effect of fasudil on NF-κB-p65 immunostaining protein expression after MTX-challenge (IHC ×400). Arrows indicate the localization and expression of NF-κB-p65 in hepatic sections. Scale bar = 200 μm. The bar graph represents the quantitative determination of NF-κB-p65 protein expression in different groups (*n* = 6); a: significant difference from the control group (*p* < 0.05); b: significant difference from the MTX group (*p* < 0.05). *** *p* < 0.001. **** *p*< 0.0001. NS, non-significant.

**Figure 7:**
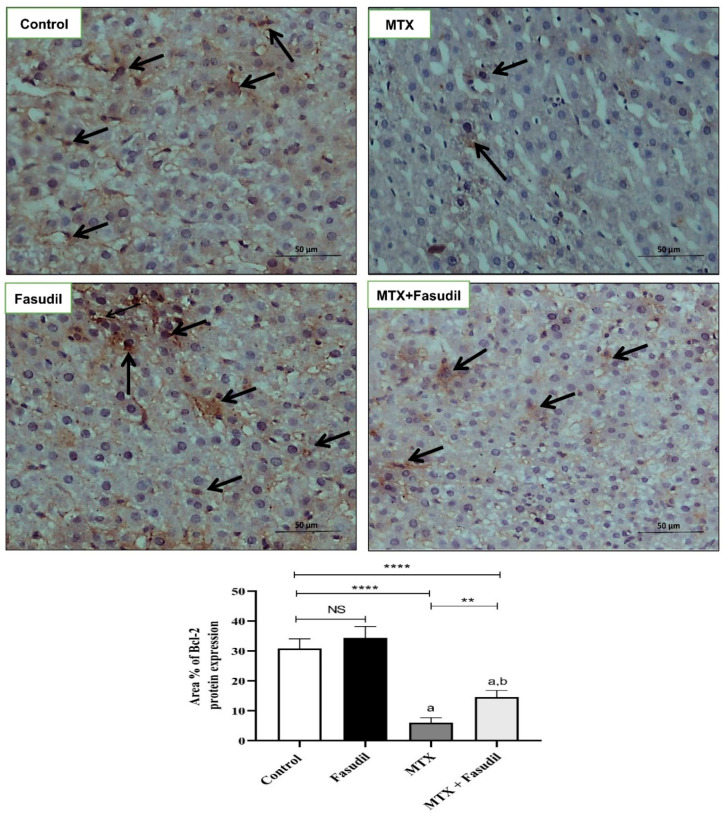
Effect of fasudil on Bcl-2 immunostaining protein expression after MTX-challenge (IHC X 400). Arrows indicate the localization and expression of Bcl-2 in hepatic sections. Scale bar = 200 μm. The bar graph represents the quantitative determination of Bcl-2 protein expression in different groups (*n* = 6); a: significant difference from the control group (*p* < 0.05); b: significant difference from the MTX group (*p* < 0.05). ** *p* < 0.05. **** *p* < 0.0001. NS, non-significant.

As shown in Figure 6, the distribution of NF-κB-p65 inside hepatic tissue using the immunostaining technique showed that MTX significantly up-regulated NF-κB-p65 expression compared to the control group, whereas fasudil i.p. administration significantly reduced NF-κB-p65 tissue up-regulation induced by MTX. On the other hand, fasudil administration didn't produce any change in the NF-κB-p65 expression compared to the control group. Moreover, hepatic tissue immunostaining for the detection of Bcl-2 distribution showed that MTX significantly down-regulated Bcl-2 distribution and expression compared to the control group. On the other hand, fasudil treatment significantly ameliorated the MTX-depleting effect on Bcl-2, and treated animals showed up-regulation of Bcl-2 compared to the MTX-treated group (Figure 7).

## 3. Discussion

Methotrexate (MTX) is a folate antagonist with immunomodulatory effects, which has been used extensively in the treatment of different autoimmune and cancerous diseases [2], but due to its severe toxicity (mainly hepatotoxicity), its clinical applications are limited. Therefore, the present study examined Rho-associated kinase (ROCK) pathway inhibition using fasudil as a new modality to attenuate MTX hepatotoxicity, which could increase MTX clinical applications and enhance patient tolerance.

MTX in low doses can induce changes in the histology of the liver, and in the long run it can produce a different type of hepatotoxicity. On the other hand, high-dose administration, as in leukemia, can result in severe hepatic tissue damage and elevation of the hepatic enzymes with progressive fibrosis and cirrhosis [10,30]. In the present study MTX prominently induced histopathological changes and hepatic tissue injury as found by histopathological examination. This was in agreement with preceding studies [8], which found that MTX can induce hepatic lesions, such as focal fibrosis, congestion, and dilatation of sinusoids with fatty vacuolation, and portal vein inflammation [31], which could be recognized, as the liver is the major site of MTX metabolism into the toxic agent 7-hydroxy-MTX, and the MTX can be stored inside hepatocytes in polyglutamated form, leading to hepatotoxicity [32,33].

On the other hand, in the present study, it was found that the i.p. administration of fasudil could significantly ameliorate the MTX injurious effect on the hepatic tissue, which could be attributed to ROCKs inhibition. Hence, ROCKs are considered the major regulator of tissue responses to injury, and fasudil, a ROCK inhibitor, and its metabolite hydroxyfasudil selectively inhibit ROCKs by competing with ATP for binding to the kinase, with minimal effects on other intracellular signaling pathways [34] in addition to antioxidant and anti-inflammatory properties [35].

The mechanism of MTX-inducing hepatotoxicity is not fully elucidated, and it can somewhat be attributed to oxidative stress generation [36]. Hence, different reports stated that MTX administration results in antioxidant enzyme depletion and free radical generation [12,37], which was in agreement with our findings. In the present study, we found that the i.p. administration of MTX significantly decreased the antioxidant enzymes SOD and catalase concentrations with depletion of hepatocellular GSH stores compared to the normal group. Hence, we used fasudil to ameliorate these effects, and we found that fasudil noticeably ameliorates the depleting effect of MTX on SOD and catalase enzymes and protected the hepatic GSH stores from diminution, assuming that Rho-ROCK pathway inhibition using fasudil has a hepatoprotective effect by amelioration of the oxidative stress inside hepatic tissue. This was in agreement with former studies, which found that the abnormal activation of the Rho/ROCK pathway was involved in different metabolic disorders, including oxidative stress, and the inhibition of Rho-ROCK pathway can diminish the oxidative stress [38].

Studies have reported that MTX administration could inhibit the cytosolic nicotinamide adenosine diphosphate (NADP)-dependent dehydrogenases and other cellular antioxidants [39,40]. NADPH is required to maintain the reduced state of cellular glutathione and protects against free radical generation, which is involved in the damage of biomolecules, such as lipids, leading to MDA upregulation [41]. In the present study, MTX significantly upregulated MDA hepatic tissue concentrations compare to the normal group, whereas the i.p. administration of fasudil remarkably attenuated the MDA upregulation by MTX, preserving the hepatocellular structure integrity, which confirms the hepatoprotective properties of fasudil. Furthermore, we found that the i.p. administration of fasudil ameliorated MTX hepatic enzyme (ALT and AST) upregulation, which was consistent with other studies’ findings [42].

RhoA can modulate several cellular functions including monocyte/macrophage chemotaxis, adhesion, and proliferation [43]. RhoA can activate the NF-κB-p65 inflammatory pathway, indicating its potential role in the inflammatory process [44]. In the present study, we examined the fasudil attenuation effect on the inflammatory process induced by MTX as a proposed hepatoprotective mechanism, and we found that fasudil could significantly attenuate the inflammatory mediators NF-κB-p65 and IL-1B upregulation produced by MTX administration, as presented by immunohistochemistry and biochemical analysis, respectively.

Researchers have found that the inhibition of the Rho/ROCK signal pathway can increase anti-apoptotic family members’ tissue concentrations [45], and the inhibition of ROCK by Y-27632 resulted in the upregulation of the anti-apoptotic protein (Bcl-2) and downregulation of the pro-apoptotic proteins [46], and it was reported that the inhibition of the ROCK pathway using fasudil decreased the number of apoptotic cells in cases of myocardial ischemic reperfusion injury [47], which agreed with our findings. Thus, we found that fasudil could significantly ameliorate Bcl 2 protein depletion by MTX, indicating the ability of fasudil to protect hepatocytes against apoptosis and degradation.

Furthermore, the present study introduced a new validated method for the detection and determination of fasudil concentration inside hepatic tissue, and we found that the MTX combined administration with fasudil significantly increased concentrations of the hepatic tissue content of fasudil compared to the fasudil only treated group, which could be attributed to decreased metabolic capacities of the liver due to MTX hepatotoxicity, leading to fasudil accumulation, increasing the fasudil protective effect by upregulating its hepatic concentrations.

## 4. Materials and Methods

### 4.1. Drugs, Reagents, and Chemicals

Fasudil and MTX were purchased from Sigma-Aldrich, St. Louis, Missouri, USA. Carbon tetrachloride (CCl4) and thiobarbituric acid (TBA) were purchased from Merck, Darmstadt, Germany. Methanol and sodium dodecyl sulfate were purchased from Thermo Fisher Scientific, Waltham, Massachusetts, USA, and Milli-Q water used to prepare buffer solutions was obtained by a Millipore^®^ purification system (Merck, Bedford, MA, USA). Other chemicals and reagents were of high analytical grade and certified sources.

### 4.2. Animals

Thirty-two adult male Sprague Dawley rats (130–150 g, 12–15 weeks) were used. Animals were kept under standard laboratory conditions of 12 h light/12 h dark cycle and 25 ± 2 °C (2 animals per cage), and they were fed standard rat chow (not less than 19% protein, 6% fiber, 3.5% fat, and 6.5% ash; EL Nasr Chemical Company, Abou-Zaabal, Cairo, Egypt) and water ad libitum. Animals were obtained from the Helwan animal breeding house, Cairo, Egypt. All animal treatments and procedures were conducted according to the ethical committee of the Faculty of Pharmacy at South Valley University (P.S.V.U 012/21), which complies with the ARRIVE guidelines and the EU Directive 2010/63/EU for animal experiments.

### 4.3. Study Design

Experimentally, rats were randomly divided into 4 groups (8 rats/group) as follows: Group I, control animals, received 0.5 mL saline/rat i.p. for seven consecutive days. Group II rats received an i.p. injection of fasudil (10 mg/kg/day) for seven consecutive days [48,49]. Group III rats received an i.p. injection of MTX (20 mg/kg) as a single dose [50,51]. Group IV rats received MTX (20 mg/kg, i.p.) as a single dose followed by fasudil (10 mg/kg/day, i.p.) for seven consecutive days.

After 24 h fasting from the last dose, rats were sacrificed (9:00 a.m.) under ketamine anesthesia (50 mg/kg, i.p.), and blood samples were collected from the inferior vena cava and cooled and centrifuged at 4000 rpm for 15 min. Sera were collected and refrigerated at −20 °C for biochemical analysis. The animals’ livers were rapidly dissected from each animal and washed using ice-cold saline, and then a portion of the hepatic tissue was taken from all animals and homogenized using 100 mmol KH_2_PO_4_ buffer solution, cooled, and centrifuged at 3000 rpm for 15 min to produce 10% homogenate. At the end of centrifugation, the supernatants were kept at −80 °C for further analysis. The remained portions of the hepatic tissues were stored in 10% formalin-buffered saline for histopathological and immunohistochemical examination.

### 4.4. Development of the Chromatographic Method

#### 4.4.1. Instrumentation

The chromatographic system consisted of a 1260 Infinity LC system (Agilent Technologies, Inc., Santa Clara, CA, USA) equipped with a binary pump and a DA detector.

#### 4.4.2. Preparation of Standard Solutions

A methanolic stock solution of fasudil was prepared to obtain a concentration of 25.0 mg/mL. Working standard solutions were freshly prepared by further dilution of the aliquots of the stock solution with the mobile phase to obtain concentrations of 1.0–12.0 µg/mL for fasudil.

#### 4.4.3. Chromatographic Conditions

The chromatographic separation of fasudil was performed using a Symmetry C18 column (150 mm × 4.6 mm, 5 μm particle size). The mobile phase consisted of a mixture of methanol/water containing 20 mM of ammonium acetate and 10 mM SDS in a 15/85 *v/v* ratio. Fasudil was eluted by an isocratic technique at a flow rate of 0.5 mL min^−1^; the column temperature was 35 °C, the detection wavelength was measured at λ = 275 nm, and the injection volume was 20 µL. Several chromatographic conditions were examined, optimized, and validated following ICH guidelines [52].

#### 4.4.4. Calibration Curve of Fasudil in Hepatic Tissue Homogenate

Appropriate volumes of working standard solution of fasudil were added to an aliquot of hepatic tissue homogenates (free from fasudil, obtained from the control animal group). The mixtures were vortex-mixed (for 3 min) and centrifuged at 6000 rpm (for 5 min) at room temperature. From the clear supernatant, 20 µL was injected into the HPLC system for analysis.

### 4.5. Histopathological and Immunohistochemical Examination

At room temperature, hepatic tissue snaps were successfully fixed in 10% formalin-buffered saline for 24 h. Liver snaps were terminated, washed, treated with different grades of alcohol, and sectioned with a thickness of 5 μm using a microtome. Then, the sections were deparaffinized using xylene and routinely stained with hematoxylin and eosin (H&E) following the standard procedures [53]. In addition, immunopositive slide sections were conducted for the immune detection of NF-κB-p65 [54] and Bcl-2 [55] according to the standard immunohistochemical procedure. Quantification of immunopositivity expression of NF-κB-p65 and Bcl-2 was conducted using ImageJ^®^ software (National Institutes of Health, Bethesda, USA) according to the previously reported investigation [56].

### 4.6. Biochemical Investigations

#### 4.6.1. Total Protein

Hepatic tissue total protein concentration was determined by the Bradford technique [57].

#### 4.6.2. Liver Enzymes

Rat sera were used for the estimation of ALT and AST concentrations using the available commercial kits (Biodiagnostics, Cairo, Egypt) following the manufacturer’s instructions [58].

#### 4.6.3. Superoxide Dismutase (SOD) Activity

The hepatic activity of SOD was measured kinetic spectrophotometrically using the SOD activity kit (Biodiagnostics, Cairo, Egypt) following the manufacturer’s protocol. Absorbance was measured at 440–460 nm using the ELISA reader.

#### 4.6.4. Catalase Activity

Samples of hepatic tissue homogenate were used for catalase activity determination using the catalase activity kit purchased from Biodiagnostics, Cairo, Egypt, and following the manufacturer’s protocol. Samples were treated using triton X-100 for solubilization of the enzyme before the assay, and the enzyme concentration was assayed using a spectrophotometric procedure based on the disappearance of hydrogen peroxide, whereas the activity of the enzyme was expressed as units/mg protein [59].

#### 4.6.5. Malondialdehyde (MDA) Content

Lipid peroxidation was determined in the hepatic tissue by measuring the MDA content. The method was a spectrophotometric assay based on the reaction between MDA and thiobarbituric acid at 95 °C in an acidic medium, producing a pink color, and its absorbance was determined at 532 nm [60].

#### 4.6.6. Proinflammatory Marker, IL-1β

The concentration of IL-1β was quantified in the collected serum using an IL-1β ELISA kit specific for rats following the manufacturer’s protocols [61].

#### 4.6.7. Statistical Analysis

GraphPad Prism software (version 9.2.0) was used for the statistical analysis of the present results. Results were expressed as means ± standard errors of the means (SE) of 8 variables. One-way analysis of variance (ANOVA) was used for statistical analysis between different groups followed by the Tukey–Kramer test to compare the mean of each group. The results were considered statistically significant when *p* ˂ 0.1.

## 5. Conclusions

In conclusion, the present study introduced fasudil as a potent hepatoprotective agent against hepatic injury produced by MTX, with underlying mechanisms as an effective anti-inflammatory (IL-1β and NF-κβ inhibition), antioxidant (SOD, catalase, and GSH upregulation), and anti-apoptotic (Bcl-2 up-regulation) drug, which may widen the clinical applications and decrease the toxicity to one of the most potent immune suppressants and anticancer drugs (MTX), increasing its therapeutic usefulness.

## Figures and Tables

**Table 1:** Summary of the HPLC method development.

Parameter	Value
λ_max_	275 nm
Retention time (min)	2.12
Linearity range (µg/mL)	1.0–12.0
LOD (µg/mL)	0.057
LOQ (µg/mL)	0.191
Regression equation	Y = a + bx
Slope	121.02
Intercept	−5.70
Correlation coefficient	0.999

**Table 2:** Accuracy results for fasudil in the standard solutions.

Standard Solution (µg/mL) (*n* = 3)	Found	% Recovery	Mean ± SD	RSD (%)
1	0.9938	99.38	0.993 ± 0.094	0.94
	0.9836	98.36		
	1.0024	100.24		
6	6.0012	100.02	6.03 ± 0.85	0.85
	6.0612	101.02		
	5.9592	99.32		
12	12.2436	102.03	12.04 ± 1.45	1.44
	11.9232	99.36		
	11.964	99.71		

SD = standard deviation; RSD = relative standard deviation.

**Table 3:** The precision of the proposed HPLC method.

Standard Solution (µg/mL)	Intra-Day Precision		Mean ± RSD	Inter-Day Precision		Mean ± RSD
	Found	% Recovery		Found	% Recovery	
1	0.9919	99.19	0.994 ± 0.30	0.9929	99.29	0.997 ± 0.57
	0.9935	99.35		1.0041	100.41	
	0.9978	99.78		0.9967	99.67	
6	5.8974	98.29	5.95 ± 0.82	5.9832	99.72	5.96 ± 0.71
	5.979	99.65		5.9154	98.59	
	5.9862	99.77		5.9928	99.88	
12	12.0372	100.31	12.07 ± 0.41	11.8764	98.97	11.98 ± 1.41
	12.054	100.45		11.9016	99.18	
	12.132	101.1		12.1812	101.51	

**Table 4:** System suitability criteria for the determination of fasudil by the HPLC method.

Parameters	FDL	Acceptable Limits
Asymmetry factor	1.06	<1.5
Tailing factor	1.25	<2
Theoretical plates (m)	4651	<2000
HETP (cm)	0.0322	

## Data Availability

Data are contained within the article.
